# Alternatives to Piglet Castration: From Issues to Solutions

**DOI:** 10.3390/ani11041041

**Published:** 2021-04-07

**Authors:** Ulrike Weiler, Maria Font-i-Furnols, Igor Tomasevič, Michel Bonneau

**Affiliations:** 1 Department of Behavioral Physiology of Livestock (460f), Institute of Animal Science, University of Hohenheim, Garbenstraße 17, 70593 Stuttgart, Germany; weiler@uni-hohenheim.de; 2 IRTA-Product Quality, Finca Camps i Armet, 17121 Monells, Spain; maria.font@irta.cat; 3 Faculty of Agriculture, University of Belgrade, Nemanjina 6, 11080 Belgrade, Serbia; tbigor@agrif.bg.ac.rs; 4 IFIP Institut du Porc, La Motte au Vicomte, 35650 Le Rheu, France

Because castrated male pigs convert feed into meat less efficiently than entire males, they are less efficient regarding the utilization of resources. Moreover, surgical castration without pain relief is painful to the piglet [1]. This is why there is a growing consensus, at least in Western Europe, that it should be abandoned [2]. There are currently three possible alternatives: surgical castration with pain relief, immunocastration, also known as vaccination against boar taint, and raising entire males.

Using anesthesia and/or analgesia during surgical castration prevents pain to the piglet. Still, this can be considered only as an intermediate solution for the short-term because it is still adverse to animal integrity, it is still resource-inefficient and it adds costs. For these reasons, surgical castration with pain relief is not sustainable for the long run in mainstream production, even if it might possibly be a solution for some production systems aiming at very high-quality products. The review papers presented in this special issue focus on the two remaining alternatives: entire males and immunocastration.

This special issue has been prepared within the framework of the cost action CA15215 “IPEMA” (Innovative approaches in pork production with entire males [3]). It contains six review papers representing the deliverables of IPEMA [4,5,6,7,8,9], a 7th review paper sponsored by IPEMA [10], and additional research papers. The present paper provides a quick summary of the issues raised by the possible alternatives to piglet castration (entire male production and immunocastration), the solutions that can be implemented now, and the knowledge that is still missing or actions that are still to be further developed. The readers are invited to read the reviews [4,5,6,7,8,9,10] for more details and references.

IPEMA organized a webinar on 15 September 2020 to share knowledge with stakeholders. At the end of the present paper, we provide the links to the various presentations prepared for the webinar, to the answers to the questions asked during the webinar, and also to a video-recording of the panel discussion that was organized during the webinar.

The main issues raised by entire males and immunocastration are summarized in Table 1.

The production of entire males raises welfare and meat quality issues.

- Entire males tend to be more aggressive and to exhibit mounting behavior, which can be detrimental to their pen mates. Because entire males are more restless than castrates, some of the farmers trying to stop castration are facing difficulties to raise them.
- The second important issue is boar taint, an unpleasant odor, and flavor that can be perceived in the meat from some entire male pigs. Two main compounds are held as responsible for boar taint: androstenone which is a testicular steroid with a urine-like odor and skatole, a product of the breakdown of the amino acid tryptophan in the hind gut, with a fecal-like odor.
- Finally there are other meat quality issues as follows:
  √ The amount of fat in entire males can be insufficient for processing dry-cured products.
  √ Because entire male pigs deposit more unsaturated fatty acids, their fat is softer and more prone to rancidity, which is again a problem for dry-cured products.
  √ Meat from entire males is less tender and has a lower water holding capacity.

All those issues in entire males come from sexual maturation. The steroids produced in the testes are responsible for better performance but also for aggressiveness and sexual behavior as well as for meat quality issues, including boar taint (Figure 1).

The following solutions to issues with entire males are available:

- Farmers’ experience and research have led to a number of solutions to the issue of aggressive and mounting behavior in entire males, including early socialization in stable groups where entire males are separated from females, provision of space in structured pens, and provision of natural materials that enrich the environment of the animal. Some farmers find it difficult to raise entire male pigs. It is very useful to facilitate experience sharing between farmers to enable those who are facing difficulties to get solutions from their colleagues. Farmers in countries that have been raising entire males for many years also have a lot of experience that could be beneficial for all.
- Preliminary results and theoretical studies indicate that selection against unwanted behaviors might be possible. Automatic recording and analysis of behaviors should help in the long run.
- Feeding pigs more saturated diets will solve the problem of excessive unsaturation of fat in many situations. But in some cases, this will not be enough and some chains, such as those supplying dry-cured products, may have to switch back to fatter pigs, either by changing breed or adapting selection goals. A still open question is the extent to which the use of fatter pigs may result in elevated incidences of boar taint.
- There are several possible solutions to the issue of toughness and lower water holding capacity:
  √ Selection for higher intramuscular fat content might contribute to reverse the lack of juiciness and tenderness. Whether the nutritional tools that are known to be efficient to increase intramuscular fat in castrates and females would also work in entire males, and what would be the resulting loss in performance, is still an open question.
  √ Use fatter animals whose carcasses will cool down more slowly in the refrigeration rooms.
  √ Feed animals more saturated diets to increase fat saturation in the carcass, which will reduce protein oxidation.
  √ Selection for higher water holding capacity is possible because this is a trait that can be measured at the slaughter line. Finding a measurable trait highly correlated with tenderness would enable selection for tenderness, but this is still to come.
  √ The extent to which it is possible to improve tenderness of entire male meat by rearing, for instance via feed restriction followed by ad libitum feeding is still an open question.
  √ Solutions at slaughter level are to reduce stress during transport and lairage and avoid mixing of pigs.
  √ Processing technologies can be adapted to accommodate the different characteristics of the meat from entire males.
  √ Ageing meat for a longer time should be beneficial for tenderness. But it is still unknown whether it is possible to age it for a sufficient time without exceeding shelf life.
- To reduce the incidence of boar taint at the farm level, selection is effective for androstenone whereas nutrition and management are effective for skatole. Low boar taint sire lines are now available. The problem is that much of the propensity of slaughter pigs to exhibit boar taint comes from the dam lines that have still to be selected against boar taint. The extent to which such a selection would be detrimental to reproductive performance is still an open question, even if the first results from research are encouraging.
- Detection of carcasses with boar taint at slaughter plant level is necessary to avoid that tainted meat reach the consumption market. The human nose has been successfully used in a number of slaughter plants across Western Europe to check boar taint on line. This method has however many weaknesses. Realistic instrumental methods that measure androstenone and skatole are coming. However, there are still uncertainties on instrumental methods regarding their total operational costs (including personnel, maintenance, and depreciation of the investment), precision and accuracy in industrial conditions (that can affect at the level of misclassified carcasses and, consequently, the risk that tainted carcasses would reach the marked or untainted carcasses would be processed as tainted) and the way to use the information they provide to establish cut-off levels. The cut-off levels should be established on the basis of the relationship between the levels of malodorous compounds and consumer acceptability, which is related to the risk of consumer dissatisfaction that the stakeholders are willing to assume. Should we aim at a robust relationship valid all across Europe or at a more precise one adapted to the distinctive characteristics of consumers in a given country or area? Is the more precise approach compatible with international trade?
- There is a growing corpus of knowledge on how to use tainted meat for processing. The first way is to reduce the quantity or concentration of the boar taint compounds. This can be achieved through a reduction of the amount of fat in the product, dilution of tainted meat with untainted meat, or cooking at high temperature. The second way is to make the perception of boar taint more difficult, by using tainted meat in products that are consumed cold or masking boar taint with smoke or spices. There are thousands of products with different combinations of fat content, processing method, serving temperature, and spicing. Testing all products to figure out their capacity to include tainted meat would be an endless task. A possible way out of this problem is to quantify more precisely the effect of each of the above-mentioned factors through modeling. Finally, the acceptability in real consumption conditions where meat is mixed with other food has been little investigated.

Immunocastration is a procedure where a vaccine is injected into the animal that inhibits steroid production by the testes. Immunocastration is effective only after the second vaccination. Before the second vaccination, animals behave and perform the same way as entire males. Immunocastration takes effect gradually after the second vaccination, some traits being affected more rapidly than others (Figure 2). Several weeks are needed for full effectiveness on all affected traits.

Unwanted behaviors decrease to very low levels within one week after the second vaccination. The advantage in performance comparatively to surgical castrates, acquired before the second vaccination, is gradually reduced with time after the second vaccination. The meat quality problems associated with entire males also decrease gradually with time. Boar taint is low within two weeks; toughness and reduced water holding capacity are also reversed quite quickly to become similar to surgical castrates; the amount of fat and unsaturation of fat takes longer to be reversed.

Welfare and meat quality issues can be raised by immunocastration, although to a lesser degree than in entire males. Vaccination may result in some stress to the animals, particularly during the second vaccination in heavier animals. This problem becomes more serious where a third vaccination is needed in very heavy animals. The same fat quality problems as in entire males can be encountered, although to a lesser extent. The longer the time interval between second vaccination and slaughter, the more immunocastrates are similar to surgical castrates for meat quality, but also for resource inefficiency. The delay between second immunization and slaughter is a convenient tool to obtain the desired meat quality characteristics achieve with a price to pay in terms of reduced performance and efficiency.

The main challenge for immunocastration in Europe is that most markets are reluctant to accept it, under the assumption that consumers may consider it as not acceptable. The results of a survey conducted within the framework of IPEMA [9] suggest that informed consumers readily accept immunocastration. A still open question is to find a smart way of informing the consumers. More generally which are the reasons why most European pork chains are reluctant to use immunocastration?

Each alternative to piglet castration without pain relief has advantages and disadvantages. The best solution for a given supply chain depends on a combination of many factors including local conditions (national regulations, farm and chain structure, local market), target products, export market, values of the chain actors, etc. Therefore there is no universal “best” alternative. Every pork chain has to find its best alternative, depending on the compromise they make between production costs and quality (or rather qualities) of the products they are selling. Concertation of all chain actors is critical to reach an agreement on the alternative to be chosen and share costs and benefits in a fair way.

All the resources made available by IPEMA can be found on the IPEMA website (http://www.ca-ipema.eu/ (accessed on 22 October 2020)). More precisely:

- The presentations given during the 15 September 2020 webinar, the video recording of the Questions and Answers session and the video recording of the panel discussion are available at https://www.youtube.com/channel/UCT-ycKszxbHoRo1RtquI_2A (accessed on 22 October 2020)
- The Working Group presentations, provided ahead of the meeting, are available at https://www.youtube.com/watch?v=tG0wJSuv3Rk&list=PL8n9r89QQpJfbuZoYQIKlmZPTmstvdglS (accessed on 22 October 2020). They include:
  √ Overview of issues and solutions, by Ulrike Weiler (University of Hohenheim, Germany) and Michel Bonneau (IFIP, France).
  √ Breeding and Genetics, by Catherine Larzul (INRAE, France).
  √ Nutrition, by Giuseppe Bee (Agroscope, Switzerland), Nathalie Quiniou (IFIP, France), Hanne Maribo (SEGES, Denmark) and Galia Zamaratskaia (University of Uppsala, Sweden).
  √ Management and Housing, by Eberhard von Borell (Martin Luther Universität Halle-Wittenberg, Germany).
  √ Innovation in grading and Meat quality control systems, by Maria Font-I-Furnols (IRTA, Spain)
  √ Innovations in the processing industry and products development, by Martin Škrlep (KIS, Slovenia).
  √ Consumer and market attitudes, by Marijke Aluwé, ILVO, Belgium.
- The complete list of questions and answers around the webinar is available on the IPEMA website at http://www.ca-ipema.eu/ (accessed on 22 October 2020).

## Figures and Tables

**Figure 1:**
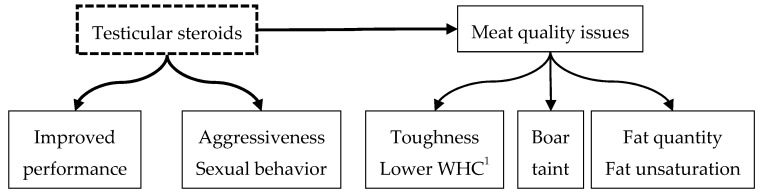
Testicular steroids are responsible for the better performance of the entire males compared to surgical castrates, but also for male-specific behaviors and lower meat quality (^1^ WHC:water holding capacity = the extent to which meat can keep its juice when maturing “drip loss” or cooking “cooking loss”).

**Figure 2:**
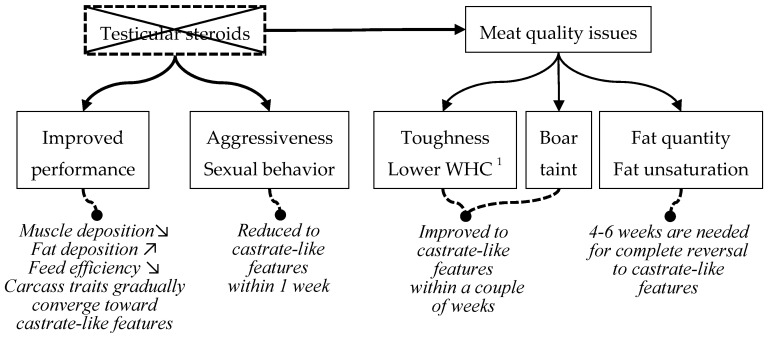
After the second vaccination, testicular steroid production is stopped, which results in dramatic changes in the metabolism of immunocastrates. Traits that are specific of entire males change to castrate-like features. Some traits are reversed more quickly than others (^1^ see Figure 1).

**Table 1:** Main issues associated with entire males and immunocastration [4,5,6,7,8,9].

Categories of Issues	Entire Males	Immunocastration ^1^
Husbandry and welfare of the animals	Aggressiveness Sexual and mounting behaviours	Stress with second or third vaccination in heavy animals
Meat quality	Boar taint Fat quantity Fat quality Toughness Water holding capacity	Same as in entire males but less and less so with increasing time interval from second vaccination to slaughter
General acceptance	May be impaired by perceived meat quality issues	Generally low in Western European supply chains ^2^

^1^ The issues associated with entire males of course apply to the animals who fail to be successfully vaccinated. ^2^ Immunocastration is widely used in Australia, New Zealand, Latin America (Brazil, Chile, Colombia, Argentina, and Mexico), Canada, and Thailand. Belgium also has a substantial production of immunocastrates.
